# Multifunctional Nanoparticles with Superparamagnetic Mn(II) Ferrite and Luminescent Gold Nanoclusters for Multimodal Imaging

**DOI:** 10.3390/polym15224392

**Published:** 2023-11-13

**Authors:** Bárbara Casteleiro, Mariana Rocha, Ana R. Sousa, André M. Pereira, José M. G. Martinho, Clara Pereira, José P. S. Farinha

**Affiliations:** 1 Centro de Química Estrutural, Institute of Molecular Sciences (IMS) and Departamento de Engenharia Química, Instituto Superior Técnico, Universidade de Lisboa, 1049-001 Lisboa, Portugal; barbara.casteleiro@tecnico.ulisboa.pt; 2 REQUIMTE/LAQV, Departamento de Química e Bioquímica, Faculdade de Ciências, Universidade do Porto, Rua do Campo Alegre s/n, 4169-007 Porto, Portugal; mariana.rocha@fc.up.pt (M.R.); arcsousa2@gmail.com (A.R.S.); 3 IFIMUP—Instituto de Física de Materiais Avançados, Nanotecnologia e Fotónica, Departamento de Física e Astronomia, Faculdade de Ciências, Universidade do Porto, Rua do Campo Alegre s/n, 4169-007 Porto, Portugal; ampereira@fc.up.pt

**Keywords:** gold nanoclusters, manganese ferrite nanoparticles, mesoporous silica, multimodal imaging, NIR-photoluminescence, superparamagnetism

## Abstract

Gold nanoclusters (AuNCs) with fluorescence in the Near Infrared (NIR) by both one- and two-photon electronic excitation were incorporated in mesoporous silica nanoparticles (MSNs) using a novel one-pot synthesis procedure where the condensation polymerization of alkoxysilane monomers in the presence of the AuNCs and a surfactant produced hybrid MSNs of 49 nm diameter. This method was further developed to prepare 30 nm diameter nanocomposite particles with simultaneous NIR fluorescence and superparamagnetic properties, with a core composed of superparamagnetic manganese (II) ferrite nanoparticles (MnFe_2_O_4_) coated with a thin silica layer, and a shell of mesoporous silica decorated with AuNCs. The nanocomposite particles feature NIR-photoluminescence with 0.6% quantum yield and large Stokes shift (290 nm), and superparamagnetic response at 300 K, with a saturation magnetization of 13.4 emu g^−1^. The conjugation of NIR photoluminescence and superparamagnetic properties in the biocompatible nanocomposite has high potential for application in multimodal bioimaging.

## 1. Introduction

Gold nanoclusters (AuNCs), with diameters below 2 nm, have been attracting a growing interest as probes for optical imaging. Unlike larger gold nanoparticles (AuNPs), with diameters above 2 nm, AuNCs have no surface plasmon resonance [1]; however, they feature size-dependent photoluminescence, large Stokes shift, high photostability and biocompatibility [2,3,4,5,6]. These characteristics make them excellent candidates for photoluminescence-based imaging [7].

Incorporation of AuNCs in multifunctional nanostructures combining photoluminescence and magnetic properties open new opportunities for developing dual bioimaging applications, combining optical imaging and magnetic resonance imaging (MRI) [7,8,9,10]. To improve MRI contrast in soft tissues, contrast agents are commonly employed, with superparamagnetic iron oxides nanoparticles already being medically approved [11,12]. The use of superparamagnetic nanoparticles, as opposed to ferromagnetic materials, is important to avoid particle aggregation. Transition metal ferrite nanoparticles (MFe_2_O_4_, with M(II) being a 3*d* transition metal cation) have high potential as MRI contrast agents due to their high saturation magnetization, easy preparation and superparamagnetic behavior at room temperature below a certain particle size [10,13]. These typically lead to negative contrast enhancement (*T*_2_-type contrast agents) [14]. Special emphasis has been put on manganese (II) ferrite MnFe_2_O_4_ because of its good colloidal stability and very high saturation magnetization values within the transition metal ferrites family [15,16,17,18].

While the conjugation of larger AuNPs and MFe_2_O_4_ nanoparticles has been widely explored in the field of catalysis [13,19,20,21,22,23,24] and multimodal imaging [25,26,27,28], the conjugation of magnetic nanoparticles with luminescent AuNCs [29,30,31,32,33,34] shows remarkable potential for multimodal bioimaging [31,32,33] and sensing [35,36].

One of the main drawbacks of most nanoparticles for multimodal systems is the lack of colloidal stability under demanding environments, such as biological media. One strategy to overcome the poor colloidal stability of AuNCs and bare ferrite nanoparticles is to use an encapsulating matrix. Among different possibilities, the condensation polymerization of alkoxysilane monomers to produce a silica matrix offers excellent opportunities for the stabilization of nanoparticles and further functionalization of the nanocomposites without affecting their properties. In the case of transition metal ferrites, the silica shell provides protection against dissolution and redox reactions in harsh media, and facilitates the conjugation with other species without interfering with the superparamagnetic behavior [13]. Silica can be prepared by simple and cost-effective routes, with good control over morphology and porosity, good colloidal stability, biodegradability and bioclearance, as well as huge flexibility for surface modification [37,38,39]. Mesoporous silica nanoparticles (MSNs) are especially promising, due to their large surface area, simple functionalization and tunable pore size [40,41,42,43,44,45]. These have been widely used in the encapsulation of different materials, such as quantum dots, carbon nanomaterials, gold nanoparticles and iron oxides. In the case of AuNCs, the encapsulation into MSNs has been mostly for application in catalysis [46,47,48,49]. Among the few examples for optical applications, AuNCs have been incorporated into a mesoporous silica shell coating Nd^3+^-sensitized up-conversion nanoparticles, for light-induced imaging-guided multifunctional cancer therapy [50].

One reason for the fact that the incorporation of AuNCs into MSNs has not been more explored is the incompatibility between the commonly used silica precursors and AuNCs [46,47,48]. Herein, we report the encapsulation of (3-mercaptopropyl)trimethoxysilane stabilized AuNCs (MPTS-AuNCs) in MSNs of 49 ± 8 nm diameter by a one-pot synthesis. This new approach allows to encapsulate the AuNCs in the silica matrix, bypassing the issues of incompatibility between the AuNCs precursors and the silica surface. This approach was further used to prepare composite nanoparticles with an MnFe_2_O_4_ superparamagnetic core, coated with a thin layer of dense silica and a mesoporous silica shell containing MPTS-AuNCs. The hybrid nanocomposite (26 ± 5 nm in diameter) features NIR emission and superparamagnetic behavior at room temperature, with a saturation magnetization of 13.4 emu g^−1^ at 300 K. The bimodal nanoparticles are prepared through green chemistry, in a simple procedure that encapsulates AuNCs and MnFe_2_O_4_ in silica without extra functionalization or ligand exchange steps.

## 2. Materials and Methods

### 2.1. Materials and Reagents

Hydrogen tetrachloroaurate(III) hydrate (HAuCl_4_·3H_2_O, ≥99.9% trace metals basis, Sigma-Aldrich, St. Louis, MO, USA), (3-mercaptopropyl)trimethoxysilane (MPTS, 95%, Sigma-Aldrich), sodium hydroxide (NaOH, pure, EKA pellets) and sodium borohydride (NaBH_4_, >98.5%, Sigma-Aldrich) were used as received in the synthesis of MPTS-AuNCs in ultra-pure water, from a Millipore Milli-Q system (resistivity ≥18 MΩ cm, Merck, Burlington, MA, USA). Absolute ethanol (99.9%, Scharlau), *N*-cetyltrimethylammonium bromide (CTAB, 99%, Sigma-Aldrich) and tetraethyl orthosilicate (TEOS, 99%, Sigma-Aldrich) were used in the synthesis of the mesoporous nanoparticles (MSNs). One-amino-2-propanol (MIPA, 93%, Aldrich), Mn(II) chloride tetrahydrate (MnCl_2_∙4H_2_O, 99%, Merck), Fe(III) chloride hexahydrate (FeCl_3_·6H_2_O, 98%, Riedel-de Haën, Seelze, Niedersachsen Germany) and hydrochloric acid (37%, analytical grade, Panreac) were used in the synthesis of the MnFe_2_O_4_ nanoparticles. Aqueous ammonia solution (NH_4_OH, 28%, VWR, Lutterworth, UK) and triethylamine (TEA, ≥99.5%, Sigma-Aldrich) were used for the fabrication of the dense and mesoporous SiO_2_ shells, respectively. All reagents were used without further purification.

### 2.2. Synthesis of (3-Mercaptopropyl)trimethoxysilane Stabilized AuNCs (MPTS-AuNCs)

Five milliliters of a 17.1 mM aqueous solution of HAuCl_4_∙3H_2_O were stirred at 30 °C under magnetic stirring. Next, 32.5 μL of MPTS were added to the solution. Finally, 100.5 μL of NaBH_4_ solution (0.11 M at 0 °C) were added dropwise. The reaction mixture was left under stirring for 5 min at 30 °C, quickly changing from yellow to a light brown color. The dispersion was used without further purification or dilution.

### 2.3. One-Pot Synthesis of MPTS-AuNCs in MSNs (MPTS-AuNCs@MSN)

The MSNs were synthesized by a modified sol–gel process [43], adapted to simultaneously incorporate the MPTS-AuNCs. In a 500 mL polypropylene flask, 47 mL of Millipore water, 0.100 g of CTAB and 350 μL of 1.08 M NaOH aqueous solution were stirred at 30 °C. Next, 1 mL of a solution of 0.13 M HAuCl_4_∙3H_2_O was added to the mixture, forming an orange precipitate. Then 175 μL of 1.08 M NaOH were added to adjust the pH to 10, and the dispersion was stirred for 2 h until the precipitate was no longer present, and a homogeneous yellow dispersion was achieved. After that, 50.5 μL of MPTS were added, leading to the change in the color of the dispersion from yellow to white. In a last step, 719 μL of NaBH_4_ solution (0.01 M in 0.22 M NaOH) were added dropwise, followed by the dropwise addition of 450 μL of TEOS. The dispersion was left stirring for 3 h at 30 °C. The final nanomaterial, MPTS-AuNCs@MSN, was purified by four cycles of washing with ethanol and centrifugation, and subsequently dried under vacuum. Finally, CTAB was removed by sonication of MPTS-AuNCs@MSN (300 mg) in an ethanolic solution of 0.5 M HCl, followed by centrifugation and drying under vacuum.

### 2.4. Preparation of MnFe_2_O_4_ Magnetic Nanoparticles (MnFe_2_O_4_ NPs)

The MnFe_2_O_4_ nanoparticles were prepared by a coprecipitation methodology previously developed by Pereira et al. [51]. For this, 10 mmol of MnCl_2_∙H_2_O were dissolved in 5 mL of an aqueous solution of HCl (2.4 M) and 20 mmol of FeCl_3_.6H_2_O were dissolved in 40 mL of water. Both solutions were heated to 50 °C and quickly mixed with 200 mL of 3.0 M aqueous solution of MIPA at 100 °C. The reaction was kept under vigorous mechanical stirring for 2 h at 100 °C. The resulting material, denoted as MnFe_2_O_4_, was magnetically separated, washed with water and stored at room temperature in aqueous medium.

### 2.5. Silica Coating of MnFe_2_O_4_ Magnetic Nanoparticles (MnFe_2_O_4_@SiO_2_)

Next, 319 μL of MnFe_2_O_4_ nanoparticle dispersions in water (21.3 g/L, MnFe_2_O_4_) were added to 80 mL of ethanol, followed by sonication for 30 min. Then 1.2 mL NH_4_OH solution (28%) were added, followed by the dropwise addition of 8 mL of 0.03 M TEOS in ethanol. The reaction was left, under stirring at room temperature for 3 h. The resulting material, denoted as MnFe_2_O_4_@SiO_2_, was washed with ethanol and dried under vacuum.

### 2.6. Hybrid Nanocomposite Conjugating MnFe_2_O_4_@SiO_2_ and MPTS-AuNCs (MnFe_2_O_4_@SiO_2_@AuNCs)

Twenty milligrams of MnFe_2_O_4_@SiO_2_ were dispersed in 7.3 mL of water. Next, 2.60 mL of 0.075 M CTAB aqueous solution were added to the dispersion, which was stirred for 10 min at 60 °C. Then, 0.2 mL of TEA were added to the dispersion. A solution of 114 μL TEOS and 1.16 mL of MPTS-AuNCs (TEOS:MPTS = 6.7 (n/n)) was added dropwise to the MnFe_2_O_4_@SiO_2_ dispersion and the resulting mixture was stirred for 2 h 30 at 60 °C. The final material, named MnFe_2_O_4_@SiO_2_@AuNCs, was washed with ethanol and dried. CTAB was removed by multiple washes with an ethanolic solution of ammonium nitrate at reflux temperature for 2 h. The characterization was performed after purification of the MnFe_2_O_4_@SiO2@AuNCs, ensuring that the silica, CTAB or AuNCs that were not incorporated in the MnFe_2_O_4_@SiO_2_ NPs were removed.

### 2.7. Characterization of the Materials

Transmission Electron Microscopy (TEM). The TEM images were acquired with two microscopes. The first one was a Hitachi transmission electron microscope (Hitachi, model H-8100, Tokyo, Japan), operating at an acceleration voltage of 200 kV, with the images being acquired by the camera KeenView of Soft Imaging System, using the software iTEM. The second TEM equipment was a JEOL JEM 1400 microscope (Peabody, MA, USA), operating at an acceleration voltage of 120 kV and equipped with a charge-coupled device (CCD) digital camera Orious (1100 W). The samples were prepared by direct deposition of 10 μL of the dispersion in a carbon-coated 400 mesh copper grid, followed by drying at room temperature.

Scanning Electron Microscopy. Energy Dispersive X-ray Spectroscopy (SEM—EDS). The SEM images were obtained on FEG-SEM JEOL JSM7001F equipment operating at 15.0 kV coupled with an EDS Inca 250 Oxford light elements detector. The samples were coated with chromium (Cr).

Confocal Microscopy and Two-photon Measurements. Confocal images were obtained on a Leica TCS SP5 (Leica Microsystems CMS GmbH, Manheim, Germany) inverted confocal microscope (DMI600). Excitation lines from an Argon ion laser or a He-Ne laser were focused into the sample by an apochromatic water immersion objective (63x, NA 1.2; Zeiss, Jena, Germany). A 111.4 μm diameter pinhole positioned in front of the image plane blocked out-of-focus signals. Two-photon excitation measurements of AuNCs were obtained using the same set-up coupled to a Ti:sapphire laser (Mai Tai, Spectra-Physics, Darmstadt, Germany) as the excitation source (wavelength range 710–990 nm, 1.7W, 100 fs, 82 MHz).

Fourier Transform Infrared (FTIR). The FTIR spectra were recorded on a Jasco FT/IR-460 Plus spectrophotometer in the 400–4000 cm^−1^ range, at room temperature, with a resolution of 4 cm^−1^ and 32 scans. The spectra of the samples were obtained using KBr pellets (Aldrich, FTIR spectroscopy grade) containing 1 wt% of the nanomaterials.

Dynamic Light Scattering (DLS). The hydrodynamic diameter of the nanomaterials was measured with a Zetasizer Nano ZS apparatus (Malvern Instruments, Malvern, UK) using laser light of 633 nm and recording the scattered light at the scattering angle of 173°.

Atomic Absorption Spectroscopy (AAS). AAS was performed using a Philips PU 9200X device (Eindhoven, The Netherlands) equipped with a hollow cathode lamp (S & J Juniper & Co, Harlow Essex, UK) to determine the concentration of the MnFe_2_O_4_ aqueous dispersion and Mn:Fe ratio. The samples were prepared by digesting MnFe_2_O_4_ aqueous dispersion with concentrated HCl (12 M). The mixture was stirred at 40 °C at room temperature overnight.

Superconducting Quantum Interference Device (SQUID). SQUID magnetometry was used to determine the magnetic properties of the samples containing MnFe_2_O_4_ nanoparticles using Quantum Design’s MPMS 3 equipment (California, USA). The measurements of the magnetization as a function of the applied magnetic field (*M*(*H*)) were performed at 300 and 5 K for a maximum applied magnetic field of 50 kOe. Temperature-dependent zero-field-cooled (ZFC) and field-cooled (FC) measurements were performed over the temperature range of 5–300 K with an applied magnetic field of 100 Oe.

UV-vis Absorption Spectroscopy. The UV-vis absorption spectra of the samples containing AuNCs were acquired on a Jasco UV-660 spectrophotometer (Jasco International, Tokyo, Japan) with a Peltier temperature controller cuvette holder (Jasco International, Tokyo, Japan).

Steady State Photoluminescence. The photoluminescence spectra of the samples containing AuNCs were recorded on a Fluorolog 3–22 spectrofluorimeter (Horiba Jobin Yvon, Irvine, CA, USA) equipped with a 450 W xenon lamp. The fluorescence quantum yields (*Φ*) were determined by the reference method using a 5,10,15,20-tetraphenyl-21*H*,23*H*-porphyrin (TPP) solution in toluene (0.24 mM; *Φ* = 11%, *λ_exc_* (excitation wavelength) = 510; 575 nm < *λ_em_* (emission wavelength) < 850 nm) as reference [52]. The quantum yield (*Φ*) was calculated as $\Phi \mathrm{sample}=\Phi \mathrm{TPP}({\mathrm{slope}}_{\mathrm{sample}}/{\mathrm{slope}}_{\mathrm{TPP}})(n_{\mathrm{sample}}^{2}n_{\mathrm{TPP}}^{2})$, where the slopes correspond to the linear correlation between the integrated emission spectra with *λ*_exc_ = 510 nm and the absorbance at *λ* = 510 nm (refractive index *n* of toluene and water equal to 1.50 and 1.33, respectively).

## 3. Results and Discussion

We prepared mesoporous silica nanoparticles (MSNs) with dual imaging capabilities, incorporating photoluminescent gold nanoclusters (AuNCs) and superparamagnetic manganese ferrite (MnFe_2_O_4_) nanoparticles. We start by developing a one-pot synthesis of MSNs incorporating AuNCs stabilized with (3-mercaptopropyl)trimethoxysilane (AuNCs@MSN) whichovercomes the incompatibility between usual preparation procedures. This was then used to develop a green encapsulation strategy for both AuNCs and MnFe_2_O_4_ nanoparticles in MSNs, without further functionalization or ligand exchange steps.

### 3.1. Synthesis and Characterization of AuNCs Stabilized with MPTS

The synthesis of MPTS-stabilized AuNCs was performed in ethanol at 30 °C, following the Brust one-phase method [53]. The Au(III) salt was solubilized in ethanol and mixed with MPTS, leading to the partial reduction of Au(III) to Au(I) induced by MPTS [54]. An aqueous solution of NaBH_4_ at 0 °C was added right after, leading to the further reduction of Au(I) to Au(0) and formation of MPTS-AuNCs. The reduction of the gold salt under mild conditions allows a better control over the AuNCs growth. The formation of AuNCs can be observed by the evolution of the color of the dispersion from yellow to brown (Figure S1A in Supporting Information). The dispersion became turbid over time, due to the hydrolysis and condensation of the methoxy groups of MPTS to form silica oligomers.

**Figure 1:**
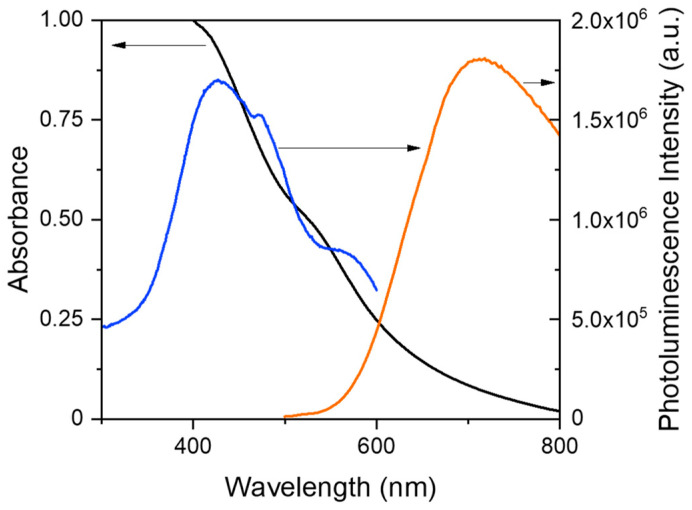
Linear optical properties of an AuNCs dispersion in ethanol (black: absorption spectrum; blue: photoluminescence excitation spectrum, *λ*_em_ = 675 nm; orange: photoluminescence emission spectrum, *λ*_exc_ = 450 nm).

Figure 1 shows the photoluminescence spectrum of a MPTS-AuNC dispersion in ethanol, featuring a broad band with maximum in the NIR (*λ*_em_^max^ = 715 nm), as well as the excitation spectrum recorded at *λ*_em_ = 675 nm (maximum intensity at *λ*_exc_^max^ = 425 nm) corresponding to the photoluminescence of the AuNCs (Figure S1B in Supporting Information).

The UV-vis absorption spectrum of the MPTS-AuNC dispersion in ethanol shows a slight shoulder at *λ* = 550 nm, corresponding to the surface plasmon resonance (SPR) of a small amount of AuNPs (with diameter above 2 nm) that are formed during the synthesis (Figure 1). The Stokes shift is 290 nm (no overlap of emission and absorption) and the photoluminescence quantum yield is *Φ* = 0.6% (calculated using TPP in toluene as reference, with excitation at λ_exc_ = 510 nm). The photoluminescence quantum yield is slightly higher than the value previously reported for AuNCs stabilized with small thiol molecules [55,56,57].

### 3.2. One-Pot Synthesis of AuNC in MSN (AuNCs@MSN)

The synthesis of the AuNCs@MSN was developed by coupling the synthesis of MPTS-stabilized AuNCs with the typical preparation method of MSNs, in water using CTAB as template and TEOS as silica precursor [37]. MPTS is a thiol-terminated organosilane, which allows the stabilization of the AuNCs with the thiol group and the incorporation of the AuNCs directly into the silica matrix through the methoxy groups. This approach overcomes the limitations previously reported in the literature, relative to the incompatibility of the silica surface with the Au(III) salt, without requiring extra steps of surface functionalization or ligand exchange [46,47,48].

The synthesis was performed in alkaline aqueous medium at 30 °C in the presence of the silica precursor. First, the Au(III) aqueous solution was added to a basic CTAB solution, producing an orange precipitate due to the complexation of HAuCl_4_ with CTAB [58]. The pH was adjusted to 10 using a 1.08 M NaOH aqueous solution. After strong stirring for 1 h, the orange precipitate was dispersed, originating a yellow colloidal suspension. After 3 h, MPTS was added to the dispersion that turned transparent due to the formation of Au(I)-MPTS complexes by the partial reduction of Au(III) to Au(I) by the thiol group of MPTS [54]. The Au reduction was completed by dropwise addition of a solution of NaBH_4_ resulting in the color change to brown, expected for the MPTS-stabilized AuNCs. Immediately after, TEOS was added dropwise to form the AuNCs incorporated in MSNs (AuNCs@MSN), which increased the turbidity and led to the flocculation of the particles (and their deposition at the bottom in the absence of stirring). This approach overcomes the difficulties previously reported for the encapsulation of AuNCs in MSNs [49], allowing the one-pot green synthesis of the nanocomposite. The AuNCs@MSN nanoparticles were then washed with ethanol and the CTAB template was removed by an HCl solution in ethanol.

**Figure 2:**
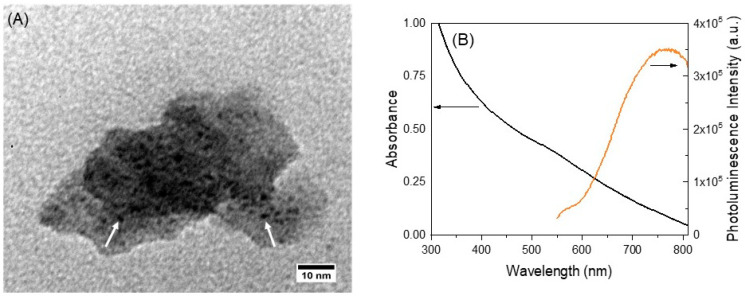
Characterization of AuNCs@MSN: (**A**) TEM image (magnification: 800,000×). (**B**) Linear optical properties in water (black: absorption; orange: photoluminescence emission spectrum, *λ*_exc_ = 300 nm).

Before purification the AuNCs@MSN hybrid particles have an average hydrodynamic diameter of 75 ± 9 nm in water (measured by DLS). However, the DLS intensity autocorrelation curve shows a noisy baseline (Figure S2 in Supporting Information), suggesting the sedimentation of AuNCs@MSN over time. Both the TEM and SEM images show that the AuNCs@MSN present an irregular shape, which can be attributed to the presence of the Au(III) salt before the formation of the MSNs (Figure 2A and Figure S3A in Supporting Information) [59]. The TEM images show that the AuNCs have an average diameter of 1.3 ± 0.2 nm and are embedded in the silica structure (Figure 2A). The SEM images taken after AuNCs@MSN purification to remove salts and free Au particles/complexes (Figure S3A in Supporting Information) yield an average AuNCs@MSN diameter of 49 ± 8 nm (Figure S3B in Supporting Information). EDS-SEM confirms the presence of Au structures in the silica matrix (Figure S3C in Supporting Information). The presence of chromium is a contamination arising from coating the sample for SEM measurements.

The UV-vis absorption spectrum of the AuNCs@MSN dispersion in water (Figure 2B) shows a weak shoulder at *λ* = 550 nm due to the SPR of AuNPs (with diameter above 2 nm) present in trace amounts (also observed for the MPTS-stabilized AuNCs described above). Upon excitation at 300 nm, a photoluminescence emission band in the NIR (*λ*_em_^max^ = 750 nm), characteristic of AuNCs, is observed (Figure 2B and Figure S4A in Supporting Information). The small red shift in the emission, compared to that observed for individual AuNCs in ethanol (Figure 1), can be attributed to the change in the AuNCs environment (silica and water), since the AuNCs@MSN are dispersed in water.

Laser excitation at 900 nm shows a quadratic dependence of the photoluminescence intensity on the excitation power, indicating that a two-photon absorption occurred (Figure S4B in Supporting Information). The silica-encapsulated AuNCs can thus be electronically excited by two-photon absorption in the NIR, which is very useful for imaging of biological samples (Figure S4C,D in Supporting Information).To better control the formation of the AuNC and MSNs, the pH was adjusted to 10 (by addition of NaOH) during both the preparation of the starting solution containing CTAB and Au(III), and the TEOS addition to form the MSNs (instead of only during the preparation of the CTAB/Au(III) solution). The NIR photoluminescence emission of the AuNCs remained, while the formation of the larger AuNPs (d > 2 nm) was suppressed, with the SPR band disappearing from the absorption spectrum of the AuNCs@MSN (Figure S5 in Supporting Information).

The influence of temperature on the morphology and photoluminescent properties of the nanomaterials was evaluated by changing the preparation temperature from 30 °C to 35 °C and 65 °C, while keeping pH 10 in the CTAB and Au(III) solutions. At 30 °C, AuNCs@MSN particles with irregular morphology are formed (Figure 2A), while at 35 °C silica rods are formed (Figure S6A in Supporting Information), and at 65 °C a mixture of worm-like and spherical particles are obtained (Figure S6B in Supporting Information). The presence of AuNPs (d > 2 nm) is more apparent in the UV-vis absorption spectra of the particles prepared at higher temperatures (Figure S6C in Supporting Information). The photoluminescence of the AuNC@MSN is slightly broadened and blue-shifted with the increase in temperature (Figure S6D in Supporting Information).

In conclusion, the best reaction temperature is 30 °C, since it is high enough for the solubilization of CTAB with the formation of aggregates making the template of the mesoporous structure, while maintaining the optical properties of the resulting AuNCs. On the other hand, the addition of NaOH during the different steps of the synthesis, opposite to the addition of NaOH only to the CTAB and Au(III) solution, seems to avoid the formation of AuNPs at lower temperature.

### 3.3. Incorporation of AuNCs and MnFe_2_O_4_ in MSNs

Magnetic nanoparticles were synthesized by coprecipitation, and their composition was determined by atomic absorption spectroscopy as MnFe_2.6_O_4_. The TEM images show nearly spherical particles with an average diameter of 13 ± 3 nm (Figure S7 in Supporting Information), similar to the results described in the literature [51].

To increase the colloidal stability and protect the nanoparticles, they were coated with a thin dense silica shell (MnFe_2_O_4_@SiO_2_). The core-shell nanoparticles have an average diameter of 15 ± 3 nm (by TEM), with their morphology unchanged (Figure S8A,B in Supporting Information). FTIR measurements (Figure S8C in Supporting Information) show that the band at 582 cm^−1^ (Fe–O and Mn–O stretching vibrations of the transition metal ferrite) is present before and after encapsulation with the silica shell. The presence of silica is confirmed by the bands at 1086 cm^−1^ with a shoulder around 1200 cm^−1^ (Si–O–Si asymmetric stretching), at 950 cm^−1^ (Si-OH stretching), 800 cm^−1^ (Si–O–Si symmetric stretching) and 464 cm^−1^ (Si–O–Si rocking) [13,60,61].

Two possibilities were explored to conjugate the MPTS-stabilized AuNCs with the MnFe_2_O_4_@SiO_2_ nanoparticles: (i) synthesis of the AuNCs simultaneously with a mesoporous silica shell (by addition of the gold salt to a dispersion containing MnFe_2_O_4_@SiO_2_, NaOH and CTAB, followed by simultaneous addition of TEOS and MPTS); and (ii) post-grafting of previously obtained AuNCs (by addition of MPTS-stabilized AuNCs to a dispersion of MnFe_2_O_4_@SiO_2_ in a solution of CTAB and NaOH, followed by the addition of TEOS). However, in both cases the nanocomposites (of a size around 30 nm by TEM) do not show magnetic properties. This is probably due to the destruction of the silica shell protecting the MnFe_2_O_4_ particles by NaOH [62]. The photoluminescence from the AuNCs in the MnFe_2_O_4_@SiO_2_@AuNCs was also very weak, without a well-defined emission band. Ammonia was then tested as an alternative base, leading to nanocomposites with a well-defined mesoporous structure and size around 35 ± 3 nm (by TEM), but without photoluminescence.

**Figure 3:**
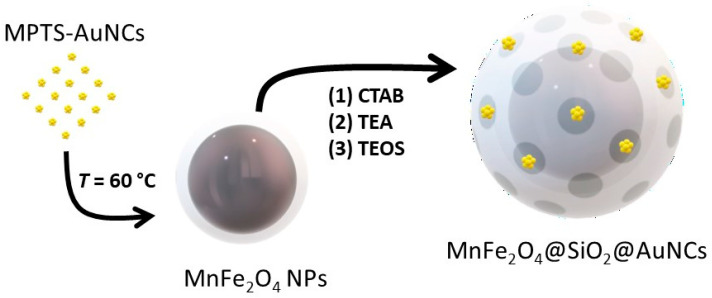
Schematics of the Mn@SiO_2_@AuNCs nanocomposite fabrication. The MPTS-AuNCs are synthesized in ethanol in a first step. Afterwards, they are mixed with Mn@SiO_2_ NPs and CTAB in an alkaline aqueous solution (TEA as base). This is followed by the addition of TEOS as silica precursor. TEA base is responsible for the hydrolysis and condensation of MPTS-AuNCs and TEOS, while CTAB induces the formation of mesoporosity in the silica network. The final nanocomposite consists of a core of MnFe_2_O_4_ with a mesoporous silica shell containing MPTS-AuNCs (Mn@SiO_2_@AuNCs).

To avoid damaging the MnFe_2_O_4_@SiO_2_ and the optical properties of the AuNCs, TEA was used as a mild base for the hydrolysis/condensation of the second layer of mesoporous silica. To accelerate the formation of the silica structure (decreasing the probability of AuNCs aggregation into AuNPs), the temperature was raised to 60 °C. In these conditions, a hybrid MnFe_2_O_4_@SiO_2_@AuNCs was obtained independently of the step in which the AuNCs were added to the MnFe_2_O_4_@SiO_2_, i.e., both for in situ and a priori synthesis of the AuNCs (Figure 3). The addition of AuNCs to an aqueous dispersion of MnFe_2_O_4_@SiO_2_ containing CTAB was performed in a second step after TEA addition, using TEOS as cross-linker between the two structures. The strategy based on a priori synthesis of the AuNCs in ethanol was found to lead to better control over the synthesis, minimizing the formation of plasmonic AuNPs (with d > 2 nm), and resulting in a more controlled preparation of the MnFe_2_O_4_@SiO_2_@AuNCs.

**Figure 4:**
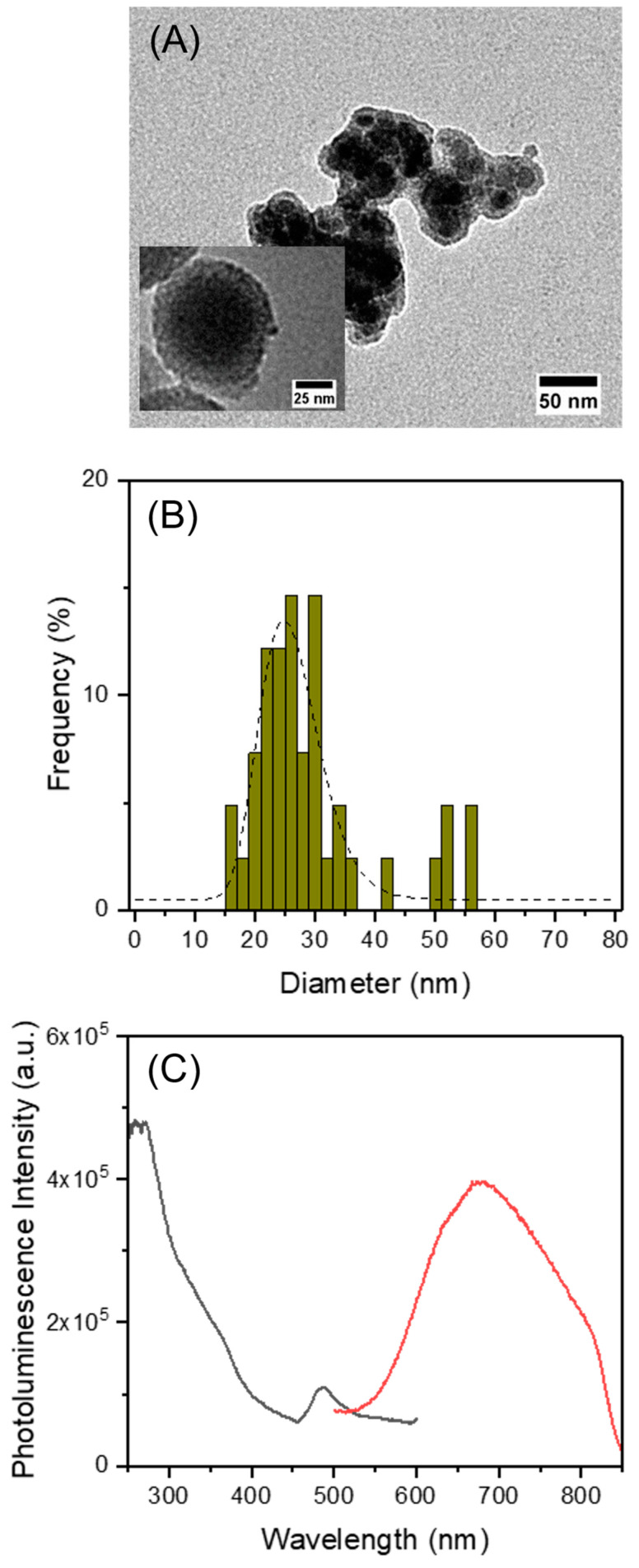
Characterization of MnFe_2_O_4_@SiO_2_@AuNCs: (**A**) TEM image (magnification: 150,000×, inset: 400,000×), (**B**) TEM particle size distribution histogram with log-normal fit (*d* = 26 nm; σ = 5 nm; R^2^ = 0.805) and (**C**) linear optical properties in water (gray: photoluminescence excitation spectrum, *λ_em_* = 675 nm; red: photoluminescence emission spectrum, *λ_exc_* = 450 nm).

The TEM image of MnFe_2_O_4_@SiO_2_@AuNCs shows the encapsulation of MnFe_2_O_4_@SiO_2_ in the mesoporous silica shell containing the AuNCs (Figure 4A). The particle size distribution determined by TEM (Figure 4B) shows that the final particles have an average diameter of 26 ± 5 nm. EDS-SEM confirms the presence of AuNCs (Au and S), and MnFe_2_O_4_ (Mn and Fe) nanoparticles (Figure S9 in Supporting Information). The shell layer was also identified by the presence of Si (the O signal can be attributed both to the MnFe_2_O_4_ and SiO_2_).

The synthesis of MSNs at *T* = 60 °C using TEA as base, in the presence of AuNCs but with no MnFe_2_O_4_@SiO_2_, leads to structures of 183 ± 55 nm in diameter by TEM. The dimensions are about six-fold higher than those obtained in similar conditions but in the presence of MnFe_2_O_4_@SiO_2_. The large dispersity in size and morphology shown by TEM (Figure S10A in Supporting Information) indicates that the MnFe_2_O_4_ nanoparticles act as nucleation sites for the mesoporous silica formation, leading to smaller structures with a lower size dispersity. Also, the synthesis of the mesoporous silica shell around the MnFe_2_O_4_ in the absence of AuNCs results in more coalesced structures (Figure S10B in Supporting Information).

Figure 4C shows the photoluminescence emission and excitation spectra of the particles, confirming the NIR emission of the AuNCs. The reported absorption spectrum of MnFe_2_O_4_ [63] overlaps with the absorption spectrum of the AuNCs, and so the amount of excitation light absorbed by the AuNCs decreases in the presence of MnFe_2_O_4_, with a consequent decrease in the AuNCs photoluminescence intensity. Nevertheless, the photoluminescence spectra of MnFe_2_O_4_@SiO_2_@AuNCs in water by excitation between 300 nm < λ_exc_ < 450 nm (Figure S11 in Supporting Information), are similar to those of the isolated AuNCs in ethanol (Figure S1 in Supporting Information).

**Figure 5:**
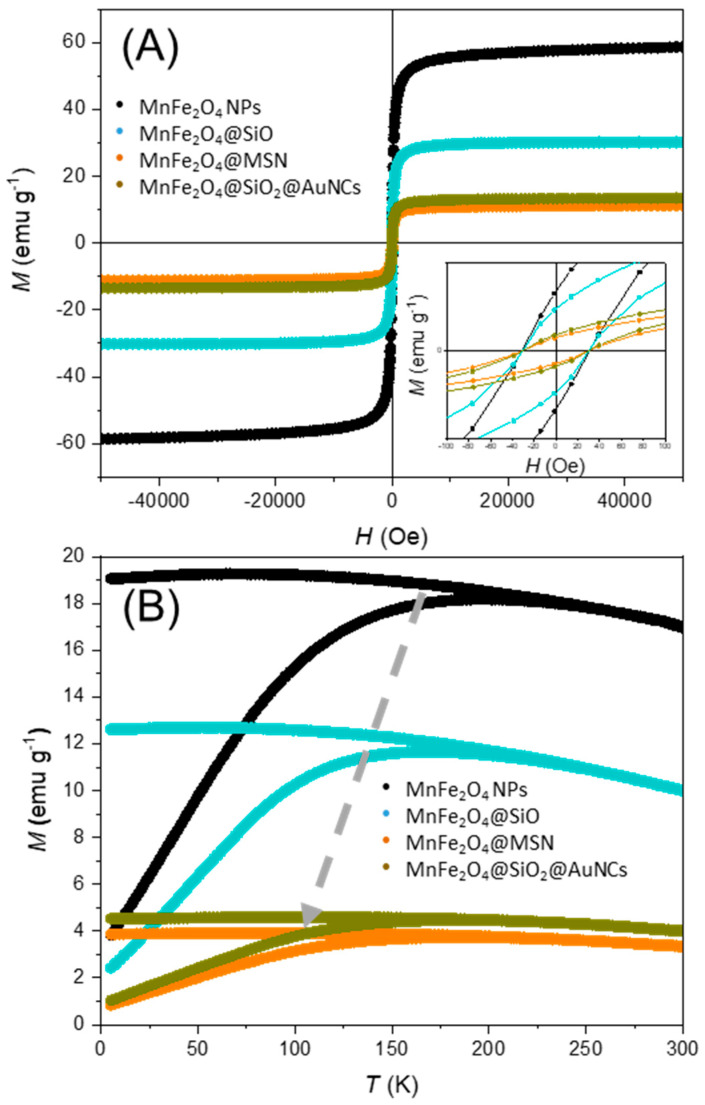
(**A**) *M*(*H*) curves between −50 and 50 kOe at 300 K (black: MnFe_2_O_4_ NPs; blue: MnFe_2_O_4_@SiO_2_; orange: MnFe_2_O_4_@SiO_2_@MSN; gold: MnFe_2_O_4_@SiO_2_@AuNCs). (**B**) Temperature-dependence of the magnetization (ZFC and FC) over the temperature range of 5–300 K with *H* = 100 Oe (black: MnFe_2_O_4_ NPs; blue: MnFe_2_O_4_@SiO_2_; orange: MnFe_2_O_4_@SiO_2_@MSN; gold: MnFe_2_O_4_@SiO_2_@AuNCs). The gray arrow indicates the evolution of the convergence point between the ZFC (lower curve) and FC (top curve) curves ongoing from the uncoated MnFe_2_O_4_ NPs to the MnFe_2_O_4_@SiO_2_@AuNCs nanocomposite.

The isothermal magnetization measured as a function of the applied magnetic field at 300 K, for the samples containing MnFe_2_O_4_ (Figure 5A) show that all samples present negligible coercivity (*H*_c_), with coercive fields around 30 Oe (Table 1), a signature of superparamagnetic behavior. The saturation magnetization (*M*_S_) values at 300 K decrease with the increase in the thickness of the silica shell, from 58.7 emu g^−1^ for MnFe_2_O_4_ to 30.1 emu g^−1^ for MnFe_2_O_4_@SiO_2_, and 13.4 emu g^−1^ for MnFe_2_O_4_@SiO_2_@AuNCs. This decrease is attributed to the diamagnetic character of silica [13]. The presence of AuNCs seems to induce a slight increase in *M*_S_, especially at 5 K (from 17.3 emu g^−1^ for MnFe_2_O_4_@SiO_2_@MSN to 19.0 emu g^−1^ for MnFe_2_O_4_@SiO_2_@AuNCs). The presence of gold has been correlated with a reduction in the magnetic disordered regions, contributing to the spin alignment at the surface, in the case of AuNPs or of a gold shell [64,65,66,67]. The magnetic behavior of AuNCs is not consensual, with in-depth studies showing that it depends on their charge, structure, size and type of ligand [68,69,70]. Nevertheless, the AuNCs are generally considered to enhance the magnetic response of the system.

The zero-field-cooled (ZFC) and field-cooled (FC) curves for the samples containing MnFe_2_O_4_ (Figure 5B) converge at the so-called reversibility temperature (*T*_rev_; see Table 1), which is lower than room temperature, indicating that the nanoparticles have superparamagnetic behavior at room temperature. However, it is not possible to estimate the blocking temperature (*T*_B_, the temperature below which the material shows ferromagnetic behavior) since the ZFC and FC curves tend to a plateau at higher temperatures for the different samples, suggesting that the dipolar interactions in MnFe_2_O_4_ are stronger than in other transition metal ferrites [51]. In the case of the silica-coated samples, the branching of the ZFC and FC curves occurs at lower temperatures (*T*_rev_), especially in the presence of the mesoporous silica shell (Table 1), indicating a decrease in the dipolar interactions between the MnFe_2_O_4_ magnetic cores, as previously reported in the literature [71].

For comparison we summarize in Table 2 the photoluminescence (*PL*) and saturation magnetization (*M*_S_) of nanocomposites of AuNCs and magnetite (Fe_3_O_4_) nanoparticles proposed for several applications. The AuNCs have been combined with Fe_3_O_4_ by electrostatic interactions [32,33,72], or through silica layers [32,36,73]. The low value of the saturation magnetization for some of the reported composites was attributed to the reduction in the magnetic properties of Fe_3_O_4_ by the coating of silica and molecular imprinted polymers [36].

The magnetic probe that is proposed herein incorporates MnFe_2_O_4_ instead of Fe_3_O_4_ magnetic nanoparticles, has higher chemical resistance and colloidal stability, good optical and magnetic properties, thus demonstrating great potential for multimodal imaging.

## 4. Conclusions

Our simple, one-pot, green synthesis of mesoporous silica nanoparticles containing photoluminescent AuNCs, uses water as solvent and mild conditions (*T* = 30 °C), overcoming the incompatibility between silica and the gold salt. The nanoparticles, with a diameter of 49 ± 8 nm, have good colloidal and optical stability over 5 months, featuring NIR emission and excellent potential for linear and non-linear photoluminescence imaging. This approach was adapted to obtain nanoparticles with both optical and magnetic response, combining a silica-coated superparamagnetic core of MnFe_2_O_4_ with a mesoporous silica shell containing the AuNCs. The preparation involves three steps: (i) synthesis of MnFe_2_O_4_ nanoparticles and coating with a thin silica shell (MnFe_2_O_4_@SiO_2_), (ii) synthesis of NIR emitting MPTS-stabilized AuNCs and (iii) conjugation of MnFe_2_O_4_@SiO_2_ and AuNCs, using CTAB as template and TEOS as silica precursor. The resulting hybrid nanoparticles, MnFe_2_O_4_@SiO_2_@AuNCs, with a 26 ± 5 nm diameter, maintain the NIR photoluminescence of the AuNPs and the magnetic properties with a saturation magnetization of 13.4 emu g^−1^ at 300 K, and superparamagnetic behavior at room temperature. The new nanocomposite with excellent optical and magnetic properties shows great potential for multimodal imaging.

## Figures and Tables

**Table 1:** Magnetic properties of the MnFe_2_O_4_-based nanomaterials: saturation magnetization (*M*_S_) at 5 K and 300 K; coercive field (*H*_c_) at 300 K; reversibility temperature (*T*_rev_).

Nanomaterial	*M*_S_ @ 5 K (emu g^−1^)	*M*_S_ @ 300 K (emu g^−1^)	*H*_C_ @ 300 K (Oe)	*T_rev_* (K)
MnFe_2_O_4_	85.4	58.7	31.0	253
MnFe_2_O_4_@SiO_2_	44.7	30.1	30.3	245
MnFe_2_O_4_@SiO_2_@MSN	17.3	11.2	30.3	217
MnFe_2_O_4_@SiO_2_@AuNCs	19.0	13.4	30.2	241

**Table 2:** Summary of nanocomposites of AuNCs and Fe_3_O_4_ NPs reported in the literature: photoluminescence (*PL*), saturation magnetization (*M*_S_) at room temperature and application.

Nanocomposite	*PL* Wavelengths (nm)	*M*_S_ (emu g^−1^)	Application	Ref.
(Fe_3_O_4_@Au@β-CD). Iron oxide-gold nanoclusters with the surface decorated with β-cyclodextrins	600–700	2.832	Bioimaging Drug Delivery	[32]
Fe_3_O_4_@AuNCs. Gold nanoclusters decorated with iron oxide NPs	650	13.0	PL Imaging MRI	[33]
Fe_3_O_4_@GSH-AuNCs. Core (iron oxide)—shell (glutathione gold nanoclusters)	468 543	29.2	Fingerprints visualization	[72]
Fe_3_O_4_@SiO_2_@AuNCs-MIP. Core (iron oxide)—shell (silica decorated with covalently bonded GSH-AuNCs) plus a molecular imprinted polymer (MIP) layer	562	9.87	Detection of Bisphenol A	[36]
Fe_3_O_4_@SiO_2_-AuNCs. Iron oxide NPs coated with a mesoporous silica shell decorated with covalently bond AuNCs	630	24	Drug delivery	[73]

## Data Availability

The data presented in this study are available on request from the corresponding author.

## Supplementary Materials

The following supporting information can be downloaded at: https://www.mdpi.com/article/10.3390/polym15224392/s1. Figure S1: Characterization of MPTS-AuNCs in ethanol: (A) Photography of the dispersion after synthesis. (B) Linear optical properties. The MPTS-AuNCs show a Stokes shift of 290 nm, with photoluminescence excitation maximum λ_exc_^max^ = 425 nm and emission maximum λ_em_^max^ = 715 nm. Similar photoluminescence emission regardless of the excitation wavelength, which is an indication of only one population of AuNCs; Figure S2: Dynamic light scattering of MPTS-AuNCs@MSN in water: (A) Autocorrelation curve. (B) Hydrodynamic diameter distribution curve by number. The autocorrelation curve shows a noisy baseline, indicating sedimentation of the MPTS-AuNCs@MSN during the measurements; Figure S3: SEM characterization of MPTS-AuNCs@MSN: (A) SEM image (magnification: 60,000×). (B) Particle size distribution (*d* = 49 ± 8 nm). (C) EDS spectrum shows the presence of Si, Au and S elements, confirming the incorporation of the AuNCs in the SiO_2_ structure; Figure S4: Optical characterization of MPTS-AuNCs@MSN in ethanol: (A) Linear optical properties (pink: UV-vis absorption spectrum; brown: emission spectrum by λ_exc_ = 300 nm; green: emission spectrum by λ_exc_ = 400 nm; purple: emission spectrum by λ_exc_ = 500 nm). (B) Power dependence of the photoluminescence intensity upon two-photon excitation at 900 nm in several regions of interest (ROI) (black: ROI 1 (slope = 2.1; R^2^ = 0.968); pink: ROI 2 (slope = 2.1; R^2^ = 0.997); blue: ROI 3 (slope = 2.0; R^2^ = 0.987)). (C) Photoluminescence emission spectra (blue: one-photon excitation λ_exc_ = 458 nm; red: two-photon excitation λ_exc_ = 900 nm). (D) Confocal image (one photon excitation λ_exc_ = 458 nm: blue; two photon excitation λ_exc_ = 900 nm: red). Blue channel corresponds most probably to the Raman scattering from the SiO_2_, while the red channel corresponds to the non-linear response of the MPTS-AuNCs in the SiO_2_ (demonstrated by the quadratic dependence of the luminescence intensity with power); Figure S5: Characterization of MPTS-AuNCs@MSN at pH = 10 adjusted using NaOH both in the starting solution containing CTAB and Au(III) and after TEOS addition: (A) TEM image (magnification: 800,000×). (B) Confocal (blue: λ_exc_ = 458 nm) and multiphoton (pink: λ_exc_ = 900 nm) image. (C) Photoluminescence emission spectrum (λ_exc_ = 300 nm). (D) One-photon absorption spectrum; Figure S6: TEM image of MPTS-AuNCs@MSN synthesized at: (A) 35 °C (magnification: 20,000×). (B) 65 °C (magnification: 40,000×). One-photon optical properties of MPTS-AuNCs@MSN (orange: 30 °C; green: 35 °C; blue: 65 °C). (C) Absorption spectra. (D) Photoluminescence emission spectra by λ_exc_ = 300 nm; Figure S7: Morphological characterization of MnFe_2_O_4_ NPs: (A) TEM image (magnification: 500,000×). (B) TEM particle size distribution histogram with log-normal fit (*d* = 13; σ = 3 nm; R^2^ = 0.992); Figure S8: Characterization of Mn@SiO_2_: (A) TEM image (magnification: 500,000×). (B) TEM particle size distribution histogram. (C) FTIR spectra of MnFe_2.6_O_4_ (orange) and Mn@SiO_2_ (blue); Figure S9: SEM-EDS characterization of MnFe_2_O_4_@SiO_2_@AuNCs: (A) SEM image (magnification: 60,000×). (B) EDS spectrum; Figure S10: TEM images of (A) Au@MSN at 60 °C using TEA as base (magnification: 9000×), (B) MnFe_2_O_4_@SiO_2_@MSN (magnification: 30,000×); Figure S11: Linear optical properties of MnFe_2_O_4_@SiO_2_@AuNCs in water (pH 10): photoluminescence excitation spectrum (λ_em_ = 675 nm) and emission spectra (300 nm < λ_exc_ < 450 nm).
