# Supplementary material for: Multifunctional Nanoparticles with Superparamagnetic Mn(II) Ferrite and Luminescent Gold Nanoclusters for Multimodal Imaging
Source: Polymers (Basel). 2023 Nov 13;15(22):4392. doi: 10.3390/polym15224392 (PMC10674285; doi:10.3390/polym15224392)
Supplement: Supplementary file 1 [file polymers-15-04392-s001.zip › polymers-2665211-supplementary.pdf]

Supporting Information

Multifunctional nanoparticles with superparamagnetic manganese(II) ferrite and gold nanoclusters for multimodal imaging

Bárbara Casteleiro,[a,b] Mariana Rocha,[b,1] Ana R. Sousa,[b,c] André M. Pereira,[c] José M. G. Martinho,[a] Clara Pereira,[b] José P. S. Farinha[a]

[a] Centro de Química Estrutural e Departamento de Engenharia Química, Instituto Superior Técnico, Universidade de Lisboa, 1049-001 Lisboa, Portugal

[b] REQUIMTE/LAQV, Departamento de Química e Bioquímica, Faculdade de Ciências, Universidade do Porto, Rua do Campo Alegre s/n, 4169-007, Porto, Portugal

[c] IFIMUP-Instituto de Física de Materiais Avançados, Nanotecnologia e Fotónica, Departamento de Física e Astronomia, Faculdade de Ciências, Universidade do Porto, Rua do Campo Alegre s/n, 4169-007, Porto, Portugal

E-mail: E-mail: jgmartinho@tecnico.ulisboa.pt; farinha@tecnico.ulisboa.pt; clara.pereira@fc.up.pt

[1] Current affiliation: IFIMUP - Instituto de Física de Materiais Avançados, Nanotecnologia e Fotónica, Departamento de Física e Astronomia, Faculdade de Ciências, Universidade do Porto, Rua do Campo Alegre s/n, 4169-007, Porto, Portugal.

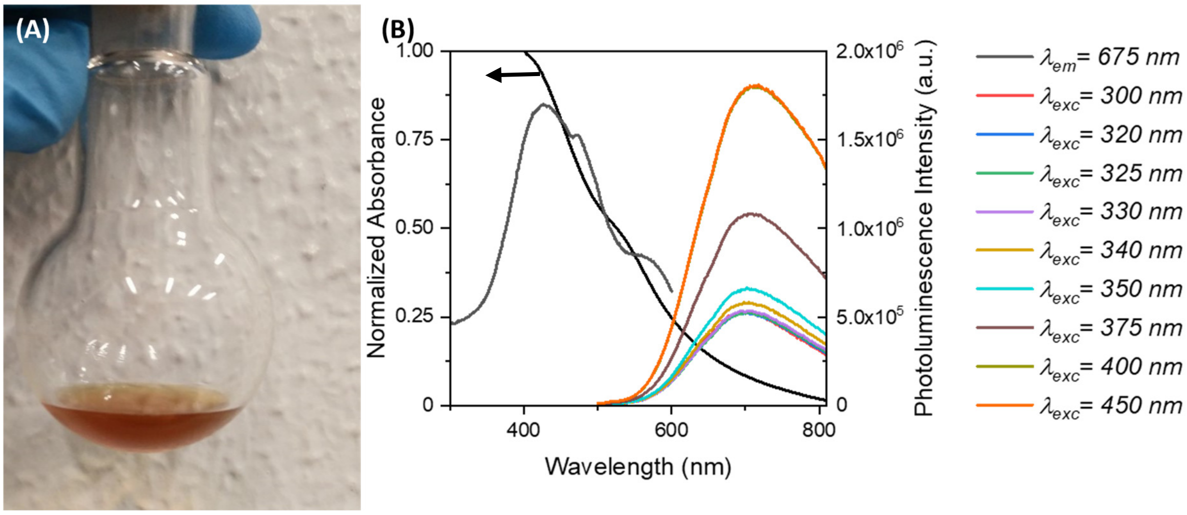

Figure S1. Characterization of MPTS-AuNCs in ethanol: (A) Photography of the dispersion after synthesis. (B) Linear optical properties. The MPTS-AuNCs show a Stokes shift of 290 nm, with photoluminescence excitation maximum λ_{exc}^{max} = 425 nm and emission maximum λ_{em}^{max} = 715 nm. Similar photoluminescence emission regardless of the excitation wavelength, which is an indication of only one population of AuNCs

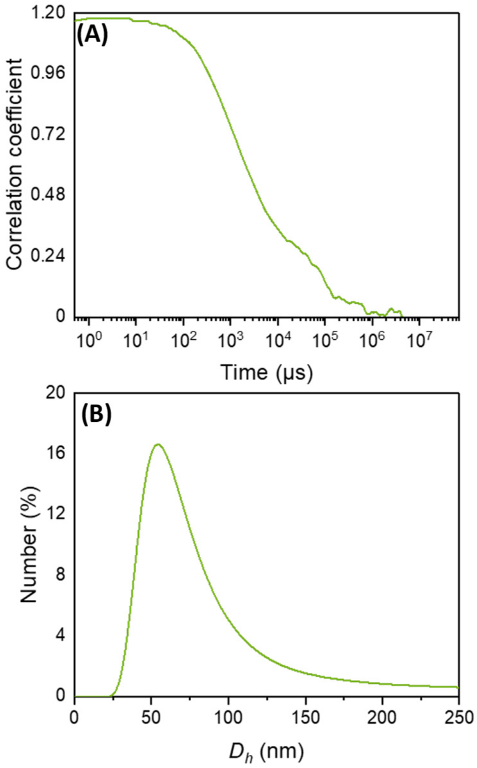

Figure S2. Dynamic light scattering of MPTS-AuNCs@MSN in water: (A) Autocorrelation curve. (B) Hydrodynamic diameter distribution curve by number. The autocorrelation curve shows a noisy baseline, indicating sedimentation of the MPTS-AuNCs@MSN during the measurements.

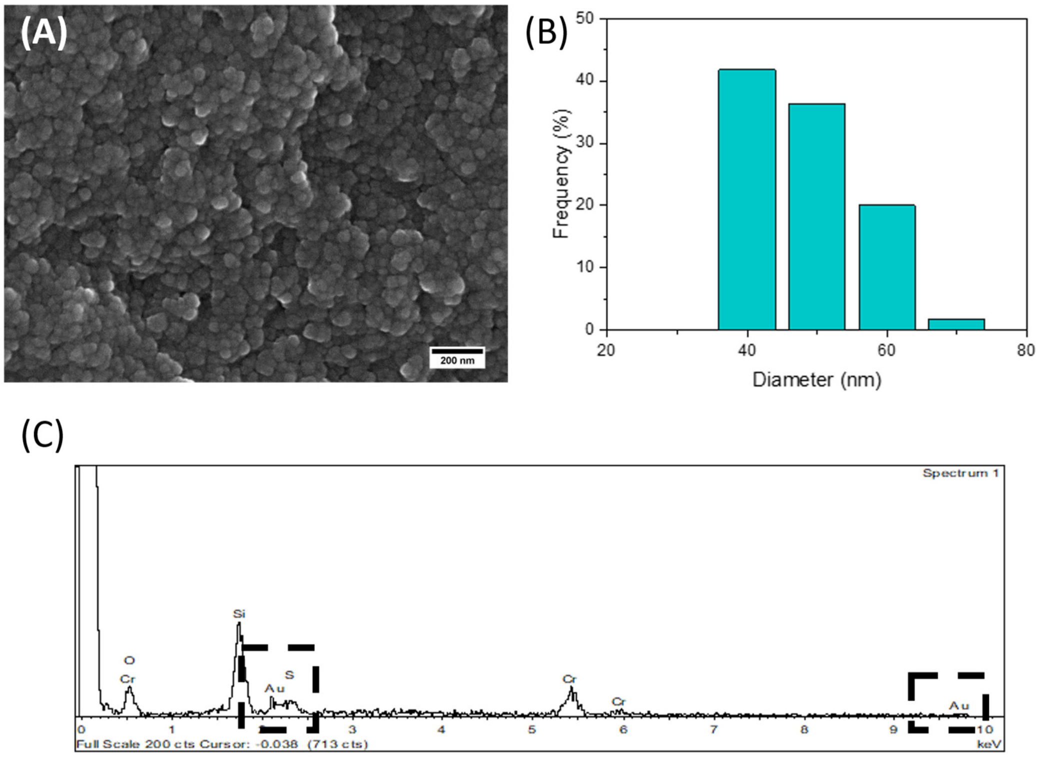

Figure S3. SEM characterization of MPTS-AuNCs@MSN: (A) SEM image (magnification: 60 000×). (B) Particle size distribution (*d* = 49 ± 8 nm). (C) EDS spectrum shows the presence of Si, Au and S elements, confirming the incorporation of the AuNCs in the SiO_2 structure.

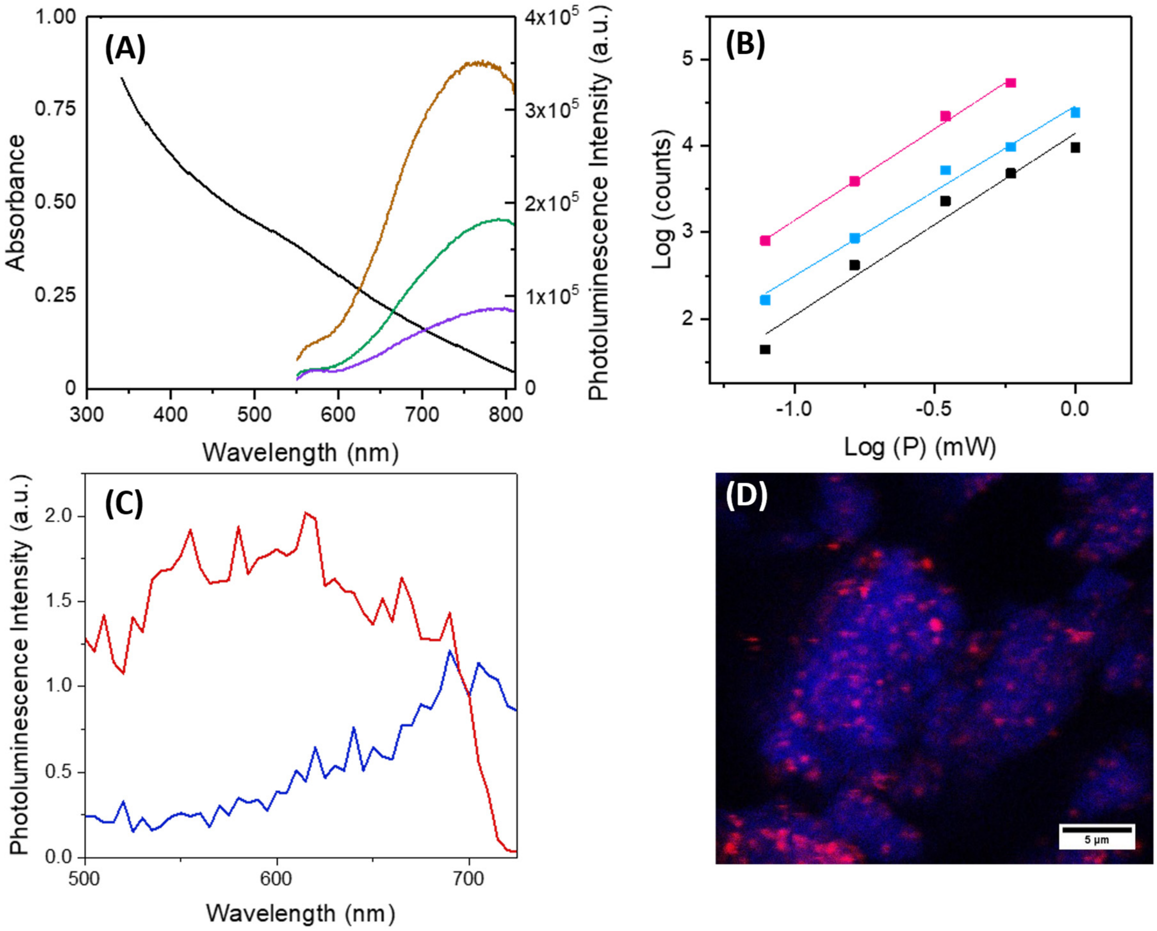

Figure S4. Optical characterization of MPTS-AuNCs@MSN in ethanol: (A) Linear optical properties (pink: UV-vis absorption spectrum; brown: emission spectrum by λ_{exc}=300 nm; green: emission spectrum *by* λ_{exc}=400 nm; purple: emission spectrum by λ_{exc}=500 nm). (B) Power dependence of the photoluminescence intensity upon two-photon excitation at 900 nm in several regions of interest (ROI) (black: ROI 1 (slope = 2.1; R^2 = 0.968); pink: ROI 2 (slope = 2.1; R^2 = 0.997); blue: ROI 3 (slope = 2.0; R^2 = 0.987)). (C) Photoluminescence emission spectra (blue: one-photon excitation λ_{exc}=458 nm; red: two-photon excitation λ_{exc}=900 nm). (D) Confocal image (one photon excitation λ_{exc}=458 nm: blue; two photon excitation λ_{exc}=900 nm: red). Blue channel corresponds most probably to the Raman scattering from the SiO_2, while the red channel corresponds to the non-linear response of the MPTS-AuNCs in the SiO_2 (demonstrated by the quadratic dependence of the luminescence intensity with power).

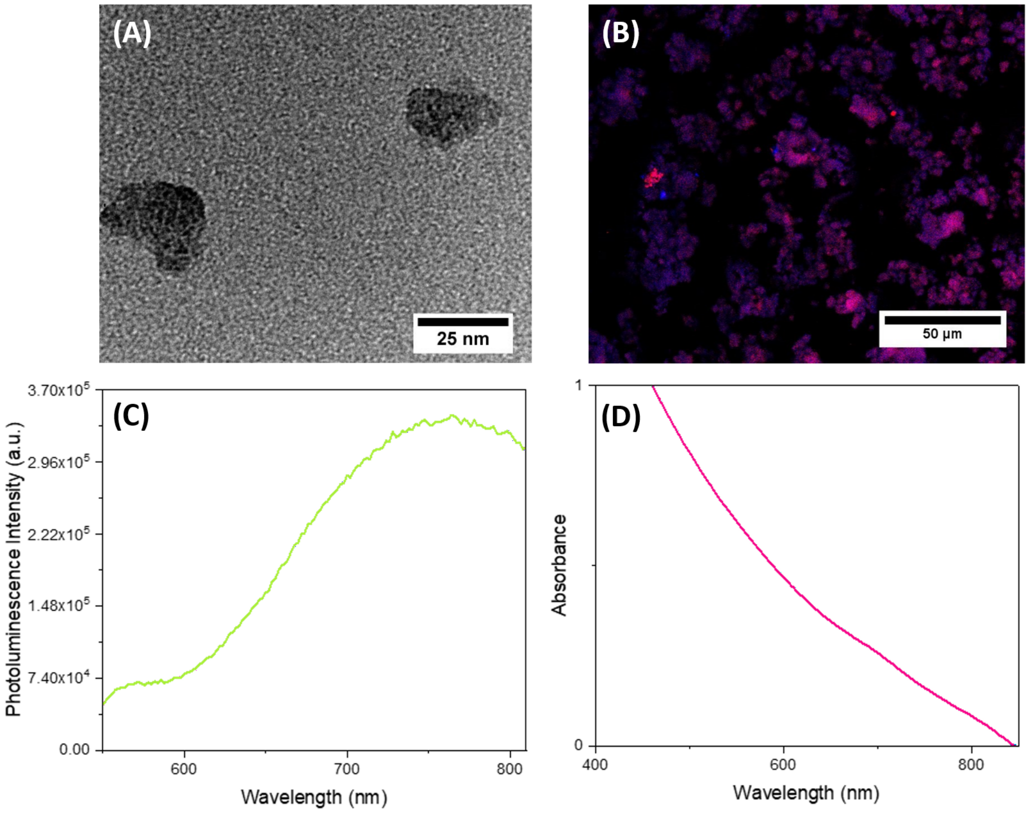

Figure S5. Characterization of MPTS-AuNCs@MSN at pH =10 adjusted using NaOH both in the starting solution containing CTAB and Au(III) and after TEOS addition: (A) TEM image (magnification: 800 000×). (B) Confocal (blue: λ_{exc} = 458 nm) and multiphoton (pink: λ_{exc} = 900 nm) image. (C) Photoluminescence emission spectrum (λ_{exc}=300 nm). (D) One-photon absorption spectrum.

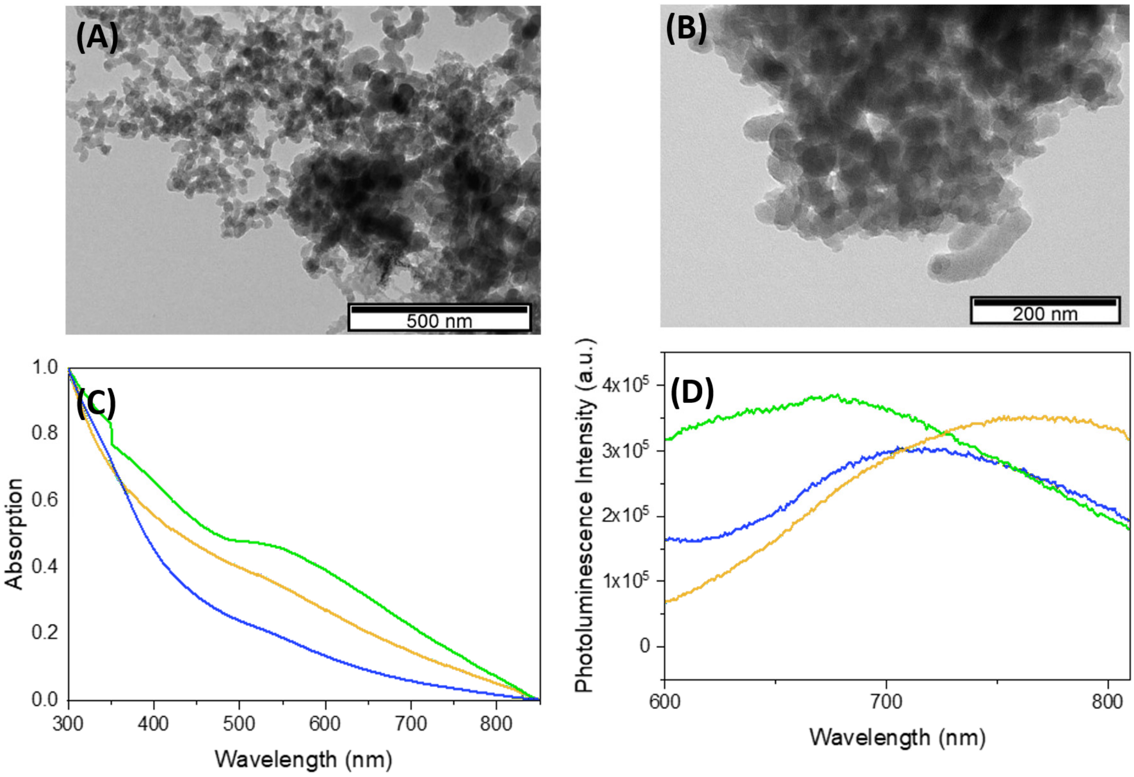

Figure S6. TEM image of MPTS-AuNCs@MSN synthesized at: (A) 35 °C (magnification: 20 000×). (B) 65 °C (magnification: 40 000×). One-photon optical properties of MPTS-AuNCs@MSN (orange: 30 °C; green: 35 °C; blue: 65 °C). (C) Absorption spectra. (D) Photoluminescence emission spectra by λ_{exc}=300 nm.

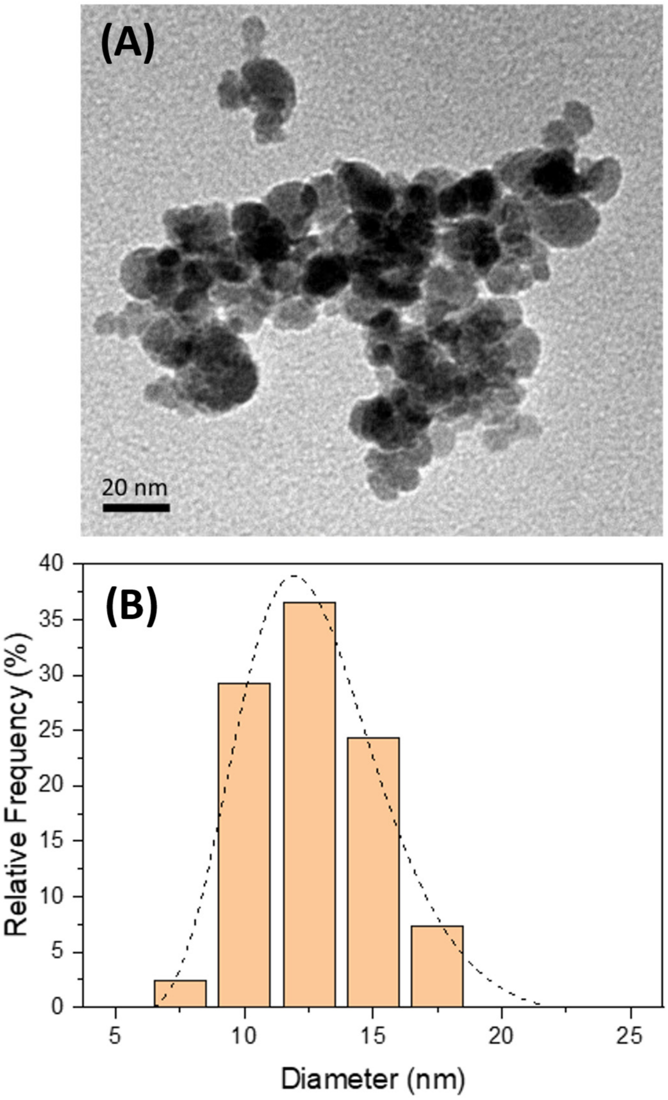

Figure S7. Morphological characterization of $MnFe_2O_4$ NPs: (A) TEM image (magnification: 500 000×). (B) TEM particle size distribution histogram with log-normal fit (*d* = 13; σ= 3 nm; R^2 = 0.992).

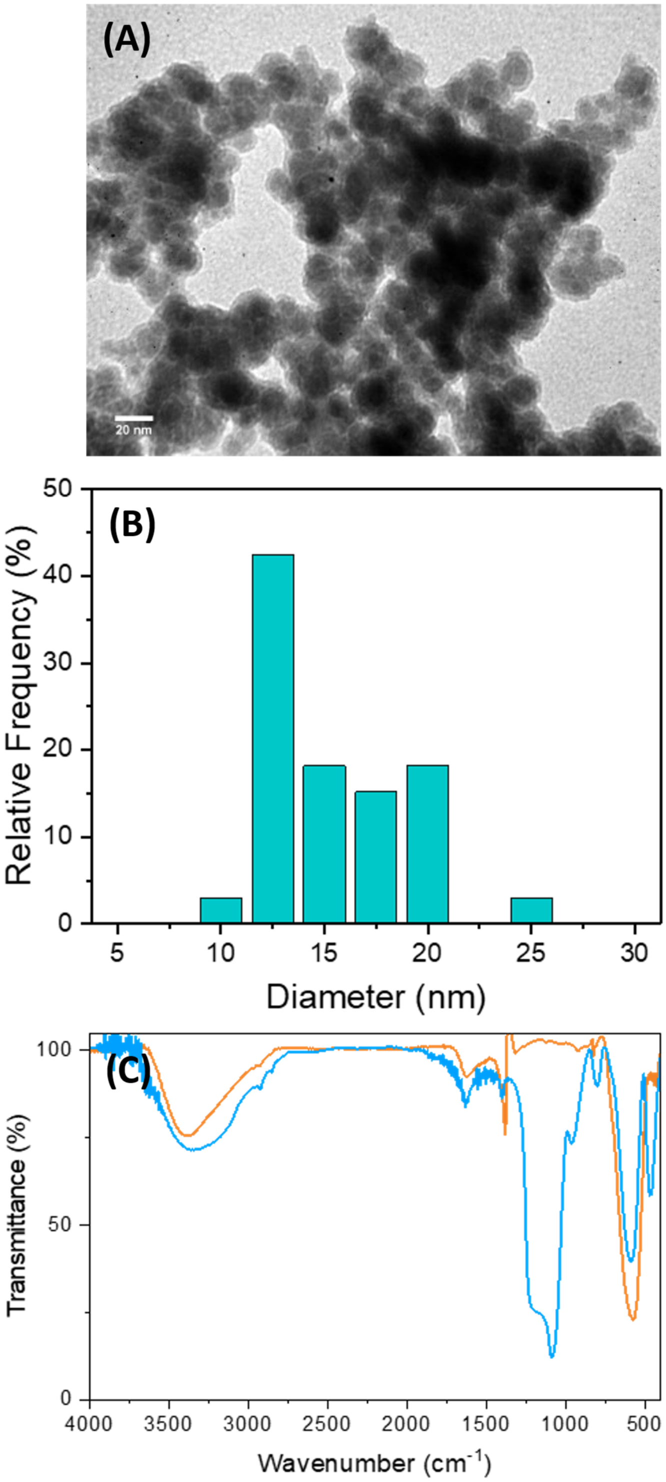

Figure S8. Characterization of Mn@SiO_2: (A) TEM image (magnification: 500 000×). (B) TEM particle size distribution histogram. (C) FTIR spectra of $MnFe_{2.6}O_4$ (orange) and Mn@SiO_2 (blue).

(A)

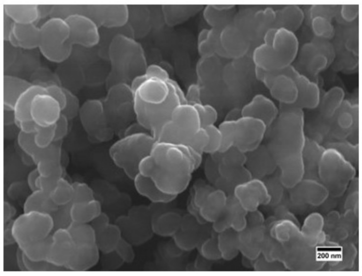

(B)

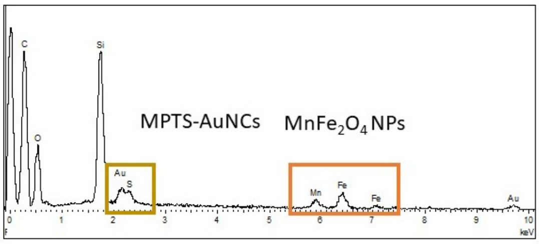

Figure S9. SEM-EDS characterization of $MnFe_2O_4$@SiO_2@AuNCs : (A) SEM image (magnification: 60 000×). (B) EDS spectrum.

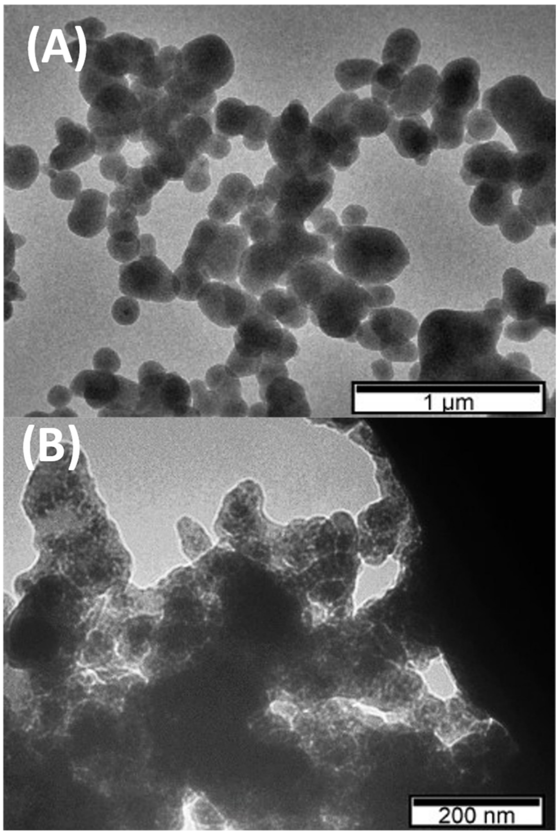

Figure S10. TEM images of (A) Au@MSN at 60 °C using TEA as base (magnification: 9 000×), (B) $MnFe_2O_4$@SiO_2@MSN (magnification: 30 000×).

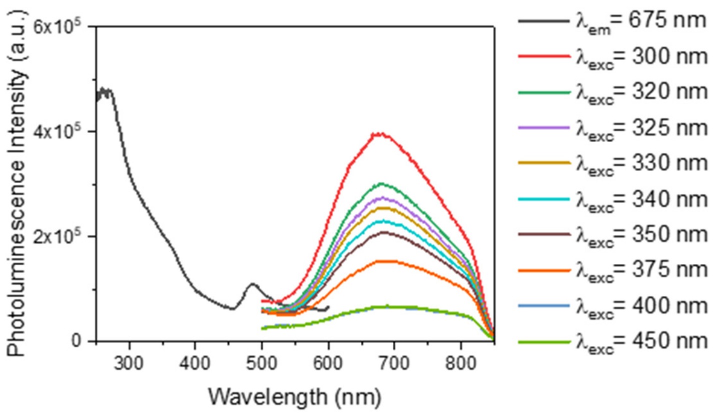

Figure S11. Linear optical properties of $MnFe_2O_4@SiO_2@AuNCs$ in water (pH 10): photoluminescence excitation spectrum (λ_{em}=675 nm) and emission spectra (300 nm < λ_{exc} < 450 nm).
